# A Real-Time Pinch-to-Zoom Motion Detection by Means of a Surface EMG-Based Human-Computer Interface

**DOI:** 10.3390/s150100394

**Published:** 2014-12-29

**Authors:** Jongin Kim, Dongrae Cho, Kwang Jin Lee, Boreom Lee

**Affiliations:** 1 Department of Medical System Engineering (DMSE), Gwangju Institute of Science and Technology (GIST), Gwangju 500-712, Korea; E-Mails: kimji@gist.ac.kr (J.K.); lightjin619@gist.ac.kr (K.J.L.); 2 School of Mechatronics, Gwangju Institute of Science and Technology (GIST), Gwangju 500-712, Korea; E-Mail: dongrae16@gist.ac.kr

**Keywords:** surface EMG, pinch-to-zoom, finger gesture recognition, machine learning, support vector machine, multi-class classification

## Abstract

In this paper, we propose a system for inferring the pinch-to-zoom gesture using surface EMG (Electromyography) signals in real time. Pinch-to-zoom, which is a common gesture in smart devices such as an iPhone or an Android phone, is used to control the size of images or web pages according to the distance between the thumb and index finger. To infer the finger motion, we recorded EMG signals obtained from the first dorsal interosseous muscle, which is highly related to the pinch-to-zoom gesture, and used a support vector machine for classification between four finger motion distances. The powers which are estimated by Welch's method were used as feature vectors. In order to solve the multiclass classification problem, we applied a one-versus-one strategy, since a support vector machine is basically a binary classifier. As a result, our system yields 93.38% classification accuracy averaged over six subjects. The classification accuracy was estimated using 10-fold cross validation. Through our system, we expect to not only develop practical prosthetic devices but to also construct a novel user experience (UX) for smart devices.

## 1. Introduction

Gesture recognition is one of the most interesting research areas because of its utility in the human computer interface (HCI) field. Systems based on visual or mechanical sensors have been commonly employed as modalities for hand and finger movement recognition [1,2]. For example, force sensitive resistors were usually used for sensing finger and hand gestures [2]. In recent years, many researchers have tried to construct a hand and finger gesture recognition system based on the surface electromyogram (sEMG), which detects the motor unit action potential (MUAP) derived from different motor units during muscle contraction [3]. Since hand and finger movement is a result of the electrical activities of muscle cells, sEMG can be used to estimate the dynamics of our hands and fingers. sEMG has the advantage of convenience and safe use on the skin because of its noninvasive characteristics [1,4,5]. Moreover, sEMG has a better signal-to-noise ratio (SNR) compared to other neural signals [1]. For these reasons, sEMG-based HCI is considered as the most practical technology among neural signal-based HCIs.

Almost all the studies on sEMG-based motion recognition have focused on arm and hand movement. For example, a study by Englehart *et al.* classified extension and flexion conditions of both arm and wrist based on wavelet analysis and principal component analysis (PCA) [6]. Englehart and Hudgins also classified four arm and wrist motions using the zero crossing rate and absolute mean value as feature vectors for a classifier [4]. Momen *et al.* constructed a real-time classification system for discriminating the various types of hand movements using sEMGs recorded from forearm extensor and flexor muscles [7]. The classification algorithm and feature vector used were the fuzzy C-means clustering algorithm and natural logarithm of root mean square value, respectively. In addition to the above studies, many researchers have tried to classify hand and arm movements using machine learning techniques such as linear discriminant analysis (LDA), artificial neural network (ANN) and support vector machine (SVM) classifier. The wavelength, Wilson amplitude, root mean square wavelet coefficients and so on are commonly used for recognizing hand and arm movement as features of classifier [8,9].

**Figure 1.**
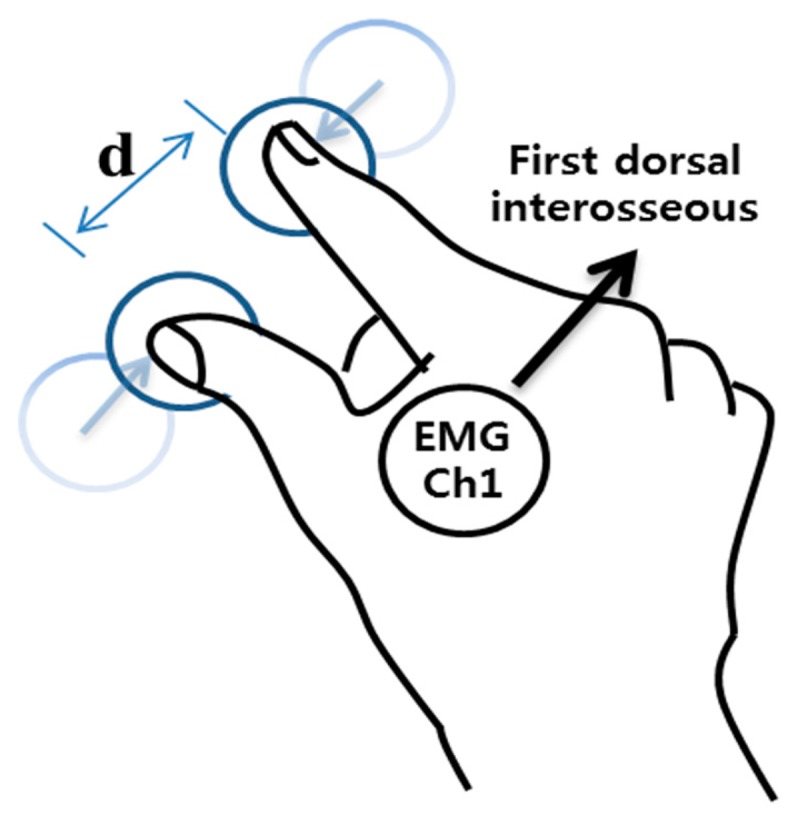
Scheme for pinch-to-zoom gesture. sEMG signal which is highly related to the pinch-to-zoom gesture is obtained from first dorsal interosseous muscle. In this figure, d means the distance between thumb and index finger.

Even though many researchers have focused on recognizing the hand movement, finger movement based on the sEMG, has also been studied because of its potential utilization in HCI and prosthetic devices. Uchida *et al.* used FFT analysis and neural networks to classify four finger motions [10]. Nishikawa *et al.* used the Gabor transform and the absolute mean value to extract the features and classify six finger motions in real time, with learning based on neural networks [11]. Nagata *et al.* used absolute sum analysis, canonical component analysis, and minimum Euclidean distance to classify four wrist and five finger gestures [12]. Chen *et al.* used mean absolute values (MAV), the ratio of the MAVs, an autoregressive (AR) model, and linear Bayesian classification to classify 5–16 finger motions [13]. Al-Timemy *et al.* used time domain-autoregression feature and orthogonal fuzzy neighborhood discriminant analysis for recognizing finger movements based on sEMG. They showed that the abduction of finger and thumb movements can be successfully classified with few electrodes [14]. Some researchers devised wearable devices such as arm- and wristbands which recognize the finger gestures. Based on their wearable systems, they developed applications to control music players, games and interpret sign language [15–17]. Although these wearable systems worked successfully, they used multiple electrodes for recognizing multiple finger gestures so they are not appropriate for real-life applications. In addition, previous studies have only concentrated on recognizing simple movements such as an extension or flexion of fingers, but there is a need to recognize more complex movements for practical applications. In our present study, we propose a real-time pinch-to-zoom gesture recognition system based on sEMG signals recorded through an electrode. Pinch-to-zoom, which is a common gesture used in smart devices, such as iPhones and Android phones, is used to control the size of images or web pages according to the distance between the thumb and index fingers (Figure 1). To infer the pinch-to-zoom gesture, we recorded sEMG signals from the first dorsal interosseous muscle and used multiclass classification techniques. Through our system, we expect to be able to not only develop practical prosthetic devices, but to also construct a novel user experience (UX) for smart devices.

The paper is organized as follows: in Section 2, we describe the configuration of the hardware and software for our system. Section 3 provides details of the experimental procedure and the algorithms used for recognizing the pinch-to-zoom gesture. Section 4 provides the results of this experiment and the interpretation of our results.

## 2. Methods and Materials

### 2.1. System Summary

**Figure 2.**
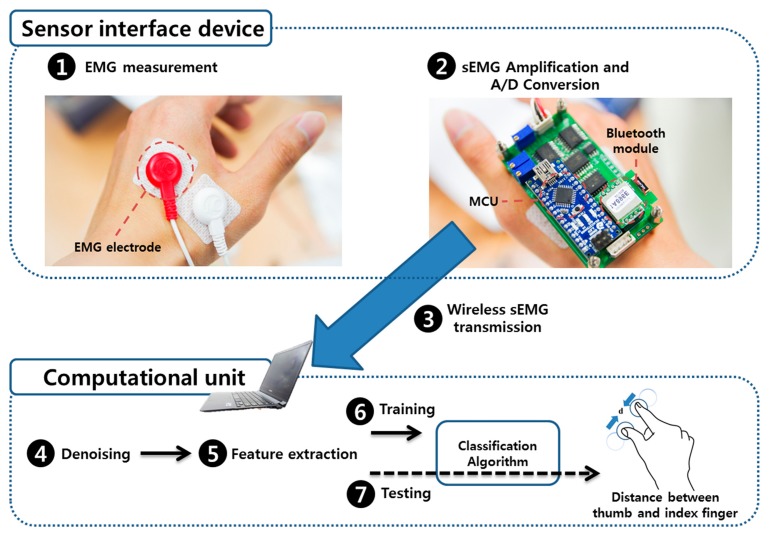
System configuration for detecting pinch-to-zoom gesture in real-time. The total system consists of sensor interface device and computational unit parts. In sensor interface device, EMG was recorded from first dorsal interosseous muscle and transmitted to computational unit parts. In computational unit, feature was extracted from sEMG and classified.

The purpose of this system is to record muscle movement using a sEMG and use it to recognize the pinch-to-zoom gesture in real time. The overall system consists of a sensor interface and computational unit parts. The sensor interface part includes a set of bipolar sEMG sensors, a microcontroller (ATmega328, Atmel Corporation, San Jose, CA, USA), and a Bluetooth module (Parani ESD-200, Sena technologies, Seoul, Korea). sEMG sensors are placed on the first dorsal interosseous muscle, which is closely related to the contraction of the thumb and index finger. The raw sEMG signal is transmitted to a computer system (Core i5, Windows 7) using bluetooth without any data loss. The software in the computational unit is developed based on Matlab (MathWorks, Natick, MA, USA). Our software provides noise reduction, feature extraction, and multiclass classification. The classification procedure is divided into training and testing sessions. The computer monitor displays instructions for finger movement during a training session. After the training session, the classifier provides a visualization of the distance between the thumb and index finger in real time. A detailed description of the 4-class classifier for this system will be provided in Section 3.4. The classifier recognizes the distance between two fingers at four levels (0 cm, 4 cm, 8 cm, and 12 cm). According to the level, the picture displayed on the computer monitor changes in real time. The overall system configuration is shown in Figure 2.

### 2.2. Software Settings

**Figure 3.**
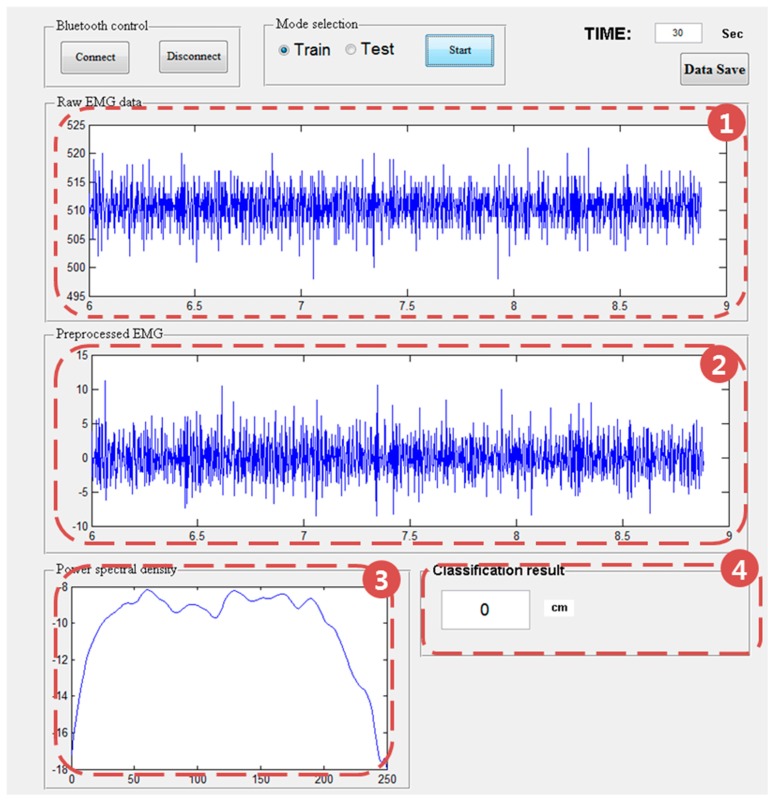
Graphic user interface (GUI) for our system. The GUI display (1) raw EMG; (2) preprocessed EMG; (3) power spectral density (PSD); and (4) the distance between thumb and index fingers.

The software was developed and implemented in Matlab for acquiring data, extracting the features, and estimating the distance between the thumb and index finger using machine learning. The following functions and tasks are performed in real time: (1) acquiring and displaying the raw sEMG data wirelessly transmitted from the sensors; (2) preprocessing the collected raw sEMG data for removing noise; (3) extracting features that are highly related to the pinch-to-zoom gesture; (4) and performing 4-class classification using a support vector machine (SVM) based on the one-versus-one (OvO) strategy. Figure 3 shows the graphical user interface for the Matlab implementation of the proposed system.

### 2.3. Subjects and Settings

Six healthy subjects (eight males and a female, mean age 27 years) were recruited among the graduate students at Gwangju Institute of Science and Technology (GIST). None of the subjects had experienced any muscular or neurological disorder that could affect our experimental results. All but one (S4) of the subjects were right-handed. Before the main experiment, a pre-test was conducted so that the subjects could familiarize themselves with the experimental protocol. All data were acquired at GIST, and a set of bipolar EMG electrodes, placed on the first dorsal interosseous muscle, was used for the EMG recording. The sampling rate was set at 1000 Hz, and all subjects were asked to sit in an armchair during recording time to prevent noise.

### 2.4. Experimental Procedure

**Figure 4.**
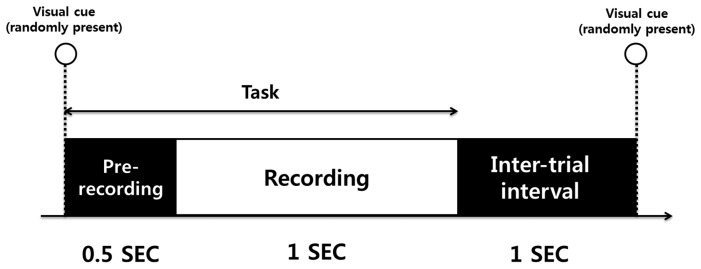
Experimental procedure. Visual cues (0 cm, 4 cm, 8 cm and 12 cm) were randomly presented during the tasks (1.5 s). Pre-recording (0.5 s) and inter-trial intervals (1 s) were also assigned.

During the experiment, our software presents four types of visual cues (0 cm, 4 cm, 8 cm, and 12 cm) to the subjects. In order to avoid the subject's prediction of the following visual cue, cue signs for 0 cm, 4 cm, 8 cm, and 12 cm were randomly displayed to the subjects though the computer monitor. All subjects were asked to perform a pinch-to-zoom gesture and maintain the distance between thumb and index finger according to the visual cue sign presented. A single trial consisted of pre-recording, recording, and an intertrial interval. A cue sign was provided for 1.5 s, and the first 0.5-s interval was reserved for gesture preparation. Only sEMG data during the recording period were used for further analysis. The intertrial interval was set to 1 s to prevent the overlap of EMG responses to successive visual cues (see Figure 4). sEMG data were acquired from 100 trials per visual cue, so a total of 400 trials per subject was used for further analysis.

### 2.5. Pinch-to-Zoom sEMG Data Analysis

**Figure 5.**
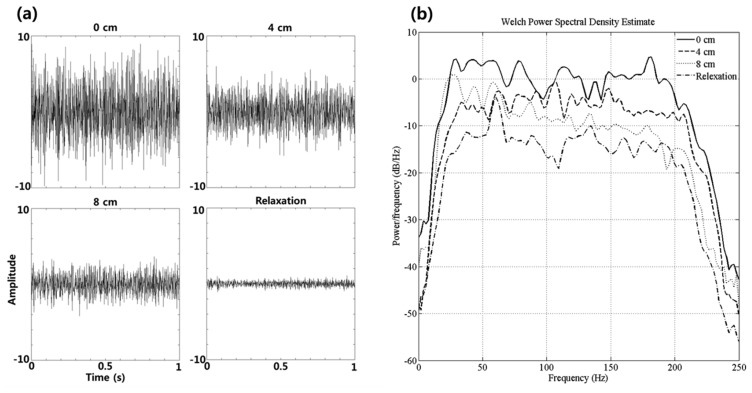
(**a**) sEMG time-series data. Amplitude of the EMG is more increased as the distance between thumb and index finger is shorter; (**b**) The power spectral density for S4. The powers in all frequency bands are statistically different (*p* < 0.01) between the four experimental conditions (0 cm, 4 cm, 8 cm, 12 cm).

As a preliminary investigation, we analyzed the statistical significance of the observed power spectrum in the four experimental conditions (0 cm, 4 cm, 8 cm, 12 cm) over all subjects. The power spectral density for each cue was estimated using Welch's method (Figure 5). Figure 5a shows that the amplitude of the sEMG which is normalized from −10 to 10 is increased as the distance between the thumb and index finger became shorter. An ANOVA test was conducted for identifying the statistically significant frequency bands. As a result, the powers in all frequency bands from 1 Hz to 250 Hz are statistically different (*p* < 0.01) between the four experimental conditions (Figure 5b). For this reason, we assumed that the powers of observed EMG data are suitable feature for recognizing the pinch-to-zoom gesture.

### 2.6. Classifier

The use of SVMs proposed by Vladimir Vapnik are a popular technique for pattern classification. The general concept of SVMs is to find the hyperplane that maximizes the margins between the nearest training points. Assume a decision hyperplane as follows [18,19]:

(1) $$\text{d}\left(\text{x}\right)={\text{w}}^{\text{T}}\text{x}+\text{b}=0$$

where **x** is a feature vector, **x** = (**x1**, …, **xd**)^T^, **w** is a normal vector of the hyperplane, and b indicates the bias. The cost function of this problem can be expressed as follows:

(2) $$\text{Maximize}\frac{2}{\left\|\text{w}\right\|}\text{subject}\;\text{to}\;\{\begin{array}{c} {\text{w}}^{\text{T}}{\text{x}}_{\text{i}}+\text{b}\geq 1,\forall {\text{x}}_{\text{i}}\in \omega_{1}\\ {\text{w}}^{\text{T}}{\text{x}}_{\text{i}}+\text{b}\leq -1,\forall {\text{x}}_{\text{i}}\in \omega_{2} \end{array}$$

where ω*_i_* is the class of sample, **x**_i_ Normal vector of the hyperplane, **w**, and bias term, b, are computed by using Equations (3) and (4):

(3) $$\text{w}=\sum_{\text{i}=1}^{\text{N}}\alpha_{\text{i}}{\text{t}}_{\text{i}}{\text{x}}_{\text{i}}$$

(4) $$\alpha_{\text{i}}({\text{t}}_{\text{i}}({\text{w}}^{\text{T}}{\text{x}}_{\text{i}}\;+\text{b})‐1)=0,\text{i}=1,\ldots ,\text{N}$$

**Figure 6.**
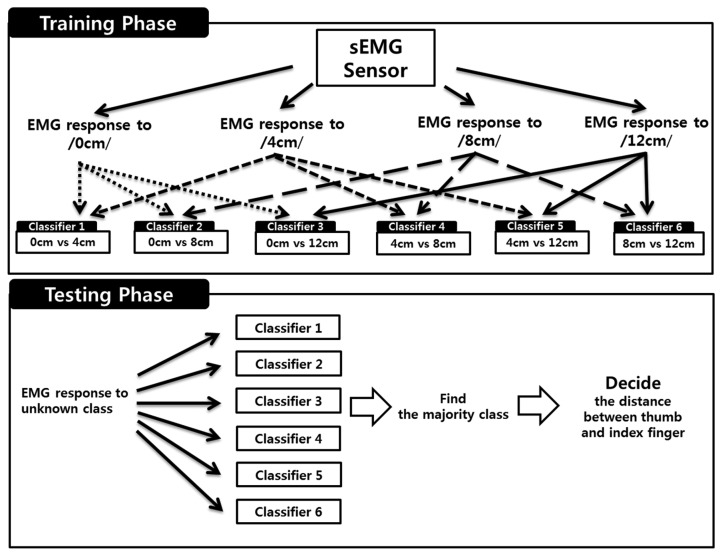
Diagram of classification algorithm for 4-class classification based on “One-Vs-One” strategy. Classification procedure consists of training phase and testing phase. In training phase, our classification algorithm trains total six binary classifiers (0 cm *vs.* 4 cm, 0 cm *vs.* 8 cm, 0 cm *vs.* 12 cm, 4 cm *vs.* 8 cm, 4 cm *vs.* 12 cm and 8 cm *vs.* 12 cm). In testing phase, sEMG response to unknown class was used for the input of six binary classifiers. The algorithms find the majority class from the outputs of six classifiers. Namely, the 4-class classification algorithm decides the majority class by the distance between thumb and index finger.

Since SVMs are basically based on two-class classification, several hyperplanes have to be used for solving an N-class problem (N > 2). In this study, we choose the OvO strategy for recognizing the pinch-to-zoom gesture. The strategy constructs one classifier per pair of classes, *i.e.*, OvO strategy trains N(N−1)/2 classifiers for a N-class classification problem. Since the number of classes, N, for our study was four (0 cm, 4 cm, 8 cm, and 12 cm), we obtained six binary classifiers using the training samples (see Figure 6).

## 3. Results and Discussion

### 3.1. Experimental Results

EMG data for a total of 400 trials per subject were used for proving the utility of our system. As preprocessing procedure commonly used for sEMG, IIR band-pass filtering was applied to all the raw EMG data (Butterworth filter, order: 4, bandwidth: 20–500 Hz). Highpass and lowpass filtering is for removing movement artifacts which is typically dominant under 10 Hz and avoiding signal aliasing which is related to high-frequency components, respectively [20]. The power spectral densities were estimated using Welch's method for feature extraction. Based on the result obtained in Section 4.1, the powers which is estimated by Welch's method were used for the feature vectors. All the data were divided into a training and a test set and only the training set was used for constructing the classifier. We repeated this procedure ten times with different random partitions for calculating the classification accuracy (10-fold cross validation). The classification accuracies for the six subjects shown in Table 1, where the highest classification accuracies among subjects are indicated in bold. The right-most column in Table 1 means the whole 4-class classification accuracy instead of just the mean of the six binary classification accuracies. Mean correct rates were always significantly higher than 91.97%. These results clearly justify the utility of our system for recognizing the pinch-to-zoom gesture in real time.

### 3.2. Discussions

Since an HCI based on sEMG interprets and transforms the action potential that is induced by the movement of muscles into control commands for computer devices, many researchers consider an sEMG-based computer interface as a natural means of HCI [1,21,22]. Most studies on gesture recognition, based on the sEMG, have focused on wrist and arm motion detection. Our present study, however, tried to recognize the finger motion using a sEMG in real-time. Unlike existing studies, which have concentrated on detecting the flexion or extension of fingers, we constructed a pinch-to-zoom gesture detection system in real time for practical applications.

Classification of sEMG responses in a single trial is very challenging because of the low SNR of the signal; therefore, signal processing techniques were required to extract task related responses from the raw sEMG signal. The overall procedure, described in our study, includes noise rejection, feature extraction, learning, and testing. First, IIR band-pass filtering was applied to the raw sEMG data for rejecting the noise. Next, we estimated the power spectral densities of filtered sEMG using Welch's method. Considering that the power of sEMG increases when a muscle is contracted, the power can be an appropriate indicator of task-related features. According to the result of Figure 5, the powers are significantly different between the four conditions (see Figure 5). Therefore, we have assumed that the powers are appropriate feature for identifying finger motor tasks.

Since an SVM was originally designed only for classifying two classes, it is necessary to construct a strategy for multiclass classification based on SVMs. In this study, we selected an OvO strategy because of its outstanding performance. The performance of our system was evaluated through 10-fold cross validation, and the mean correct rate over all subjects was 93.38% for 4-class classification. All experiments were conducted in Matlab.

In order to construct a myoelectric interface for real-life use, some critical issues should be considered. First, we should consider that most myoelectric interfaces are not appropriate for multi-user situations because sEMG signals are user-dependent. Since the skin impedance, thickness of subcutaneous fat, and the way muscles are moved for same gesture differ considerably among users, different classifiers have to be trained for individual users. This inconvenience of standard myoelectric interfaces makes them impractical, therefore, it is necessary to design a myoelectric interface for multiple users [23–27]. In our present study, we also tested the classification performance of our system for multiple users. We used the sEMG signal of a subject as test set, and the sEMG signals of remaining subjects as training set. We repeated this process for all subjects, and derived averaged classification accuracy. As a result, the averaged recognition rate was 41.36% ± 3.43%. Although this result is much over chance level for four-class classification, it is not enough for real-life application. Therefore, in the future study, we will develop the novel algorithm such as bilinear modeling in order to extract the user independent factors from sEMG signals for multi-user interfaces [28].

The second problem which has to be solved for practical application is the displacement of the electrodes. For recognizing the gesture using a sEMG-based system, it is necessary to acquire the task-related sEMG signal on a consistent muscle during training and testing. If electrodes are placed in the wrong position, the performance of the classifier may decline significantly. However, in the case of finger gesture recognition, it is very challenging to place the electrodes on exactly the same muscles since the muscles related to finger movements are usually very small. In this study, we recorded sEMGs on the first dorsal interosseous muscle, which is located between the thumb and index finger. Since the first dorsal interosseous muscle is the largest and strongest among the dorsal interosseous muscles, it can be easily found for all subjects and the SNR of the sEMGs recorded from the first dorsal interosseous muscle is better than the SNR of sEMGs recorded from the other dorsal interosseous muscles. When the distance between thumb and index finger become minimized, this muscle is maximally contracted and becomes swollen; therefore, we can easily find the specific location of the first dorsal interosseous muscle. This means that by using the sEMGs recorded from the first dorsal interosseous muscle, we can conveniently acquire pinch-to-zoom gesture-related sEMG signals from a consistent muscle for all subjects.

Another obstacle for a practical application is how to select the appropriate number of classes. Since the number of classes and classification performance for a classifier is a trade-off, myoelectric devices usually recognize the gesture as two classes such as extension and flexion. Even though this approach shows good classification performance in a laboratory environment, two classes are not enough for real applications. Our study classified pinch-to-zoom gesture into four classes (0 cm, 4 cm, 8 cm and 12 cm). Although four classes may be still not enough to recognize smooth pinch-to-zoom gestures, it is not imperative to recognize the smooth pinch-to-zoom for practical applications, so that we choose only four distinct classes which show a high classification rate. However, in future study, we will try to construct the system to recognize the pinch-to-zoom gesture as more classes than four with high classification rates.

**Figure 7.**
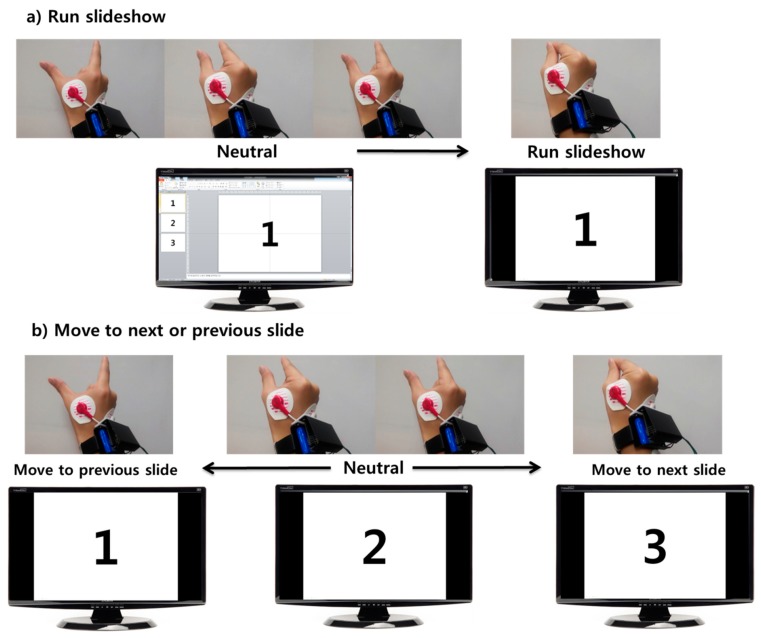
Snapshots of the application to control Powerpoint 2010 based on the pinch-to-zoom recognition system. (**a**) Scenario to run a slideshow. In this case, our system transforms the result of classifier, 0 cm into the command, “run slideshow” and the others (4 cm, 8 cm and 12 cm) into neutral commands; (**b**) Scenario to move slide. In this case, our system transforms the 12 cm result of the classifier into the command, “move to previous slide”, 0 cm into “move to next slide”, and both 4 cm and 8 cm into neutral.

As a practical application, we developed the software to control a presentation program (Powerpoint 2010, Microsoft, Redmond, WA, USA) based on our system. In this application, the results of the classifier (0 cm, 4 cm, 8 cm and 12 cm) are transformed into the commands, “run slideshow”, “move to previous slide”, “move to next slide”, and neutral (see Figure 7). We used this tool for a presentation during 20 min without any errors. It shows that our system can be used in real-life applications. In addition, since the first dorsal interosseous muscle is highly related to pinch-to-zoom gestures as well as clicking motions, our system can be also used for recognizing the clicking motion which implies the tapping of index fingers. Therefore, our system was successfully utilized for the presentation software based on clicking motion with the same hardware and software. In this system, when subjects tap their index finger, the presentation program moves to the next slide.

Considering the superior classification accuracy and low computational load, we expect that this system can be used in many types of applications, such as smart device control, robot arm control, sign language recognition, and game applications. For example, the system allows users to control web browsers or video actions of smart phones without touching the screen. Furthermore, this system has huge potential as a game controller because the video game industry requires quick and intuitive interfaces that can be used as game controllers. Existing devices have many physical buttons that require a lot of effort to master. Our system, however, can directly transform the movement of a user to the movement of a character in a video game. This could provide a new gaming experience to the users. Another important application of the system would be to translate sign language for the speech-impaired. Based on the remarkable classification accuracy, we expect to develop an outstanding sign language recognition system.

## 4. Conclusions/Outlook

In summary, it is possible to recognize a pinch-to-zoom gesture based on sEMG that is recorded on the first dorsal interosseous muscle. For the resulting multiclass classification problem, we used an OvO strategy based on SVMs. This system demonstrates outstanding classification accuracy and runs in real time. In comparison with existing studies on finger motion detection, our system recognizes a more complex gesture like pinch-to-zoom, so we expect this system to be usefully employed in many applications such as smart device control, robot arm control, sign language recognition, and game controllers.

## Figures and Tables

**Table 1.** Classification accuracies in % for classifying test trials.

**Subject**	**0 cm *vs.* 4 cm**	**0 cm *vs.* 8 cm**	**0 cm *vs.* 12 cm**	**4 cm *vs.* 8 cm**	**4 cm *vs.* 12 cm**	**8 cm *vs.* 12 cm**	**4-Class Classifying Accuracy**
S1	80.42 ± 13.44	100	100	100	100	98.89 ± 3.51	88.49 ± 6.56
S2	97.64 ± 4.99	98.75 ± 3.95	100	93.89 ± 6.45	100	100	93.24 ± 5.40
S3	100	100	100	88.06 ± 12.34	100	100	95.11 ± 3.79
S4	92.92 ± 8.06	98.75 ± 3.95	100	100	100	100	98.16 ± 4.18
S5	80.83 ± 18.77	96.39 ± 5.83	100	96.39 ± 5.83	100	100	89.56 ± 7.81
S6	100	100	100	96.25 ± 6.04	95.14 ± 6.29	100	95.74 ± 2.95
Mean Correct Rate	91.97 ± 9.16	98.98 ± 1.4	100	95.77 ± 4.46	99.19 ± 1.98	99.81 ± 0.45	93.38 ± 3.73
